# Association between gut permeability, brain volume, and cognition in healthy participants and patients with schizophrenia spectrum disorder

**DOI:** 10.1002/brb3.3011

**Published:** 2023-04-24

**Authors:** Toon Anton Willem Scheurink, Jenny Borkent, Shiral S. Gangadin, Sahar El Aidy, Rene Mandl, Iris E. C. Sommer

**Affiliations:** ^1^ Department of Biomedical Sciences of Cells & Systems University of Groningen, University Medical Center Groningen Groningen The Netherlands; ^2^ Host‐Microbe Metabolic Interactions Groningen Biomolecular Sciences and Biotechnology Institute (GBB) University of Groningen Groningen The Netherlands; ^3^ Department of Psychiatry University of Groningen University Medical Center Groningen Groningen The Netherlands

**Keywords:** bacterial translocation, brain volume, microbiome‐gut‐brain axis, MRI, schizophrenia spectrum disorders

## Abstract

**Introduction:**

The barrier function of the gut is important for many organs and systems, including the brain. If gut permeability increases, bacterial fragments may enter the circulation, giving rise to increased systemic inflammation. Increases in bacterial translocation are reflected in higher values of blood markers, including lipopolysaccharide binding protein (LBP) and soluble cluster of differentiation 14 (sCD14). Some pioneer studies showed a negative association between bacterial translocation markers and brain volumes, but this association remains scarcely investigated. We investigate the effect of bacterial translocation on brain volumes and cognition in both healthy controls and patients with a schizophrenia spectrum disorder (SSD).

**Materials and methods:**

Healthy controls (*n* = 39) and SSD patients (*n* = 72) underwent an MRI‐scan, venipuncture and cognition assessments. We investigated associations between LBP and sCD14 and brain volumes (intracranial volume, total brain volume, and hippocampal volume) using linear regression. We then associated LBP and sCD14 to cognitive function using a mediation analysis, with intracranial volume as mediator.

**Results:**

Healthy controls showed a negative association between hippocampal volume and LBP (*b* = –0.11, *p* = .04), and intracranial volume and sCD14 (*b* = –0.25, *p* = .07). Both markers were indirectly associated with lower cognitive functioning in healthy controls (LBP: *b* = –0.071, *p* = .028; sCD14: *b* = –0.213, *p* = .052), mediated by low intracranial volume. In the SSD patients, these associations were markedly less present.

**Conclusion:**

These findings extend earlier studies suggesting that increased bacterial translocation may negatively affect brain volume, which indirectly impacts cognition, even in this young healthy group. If replicated, this finding stresses the importance of a healthy gut for the development and optimal functioning of the brain. Absence of these associations in the SSD group may indicate that other factors such as allostatic load, chronic medication use and interrupted educational carrier had larger impact and attenuated the relative contribution of bacterial translocation.

## Graphical abstract

*LEFT*: Healthy controls (*n* = 39) and SSD patients (*n* = 72) underwent an MRI‐scan, venipuncture and cognition assessments. We investigated associations between LBP and sCD14 and brain volumes (intracranial volume, total brain volume and hippocampal volume) using linear regression. We then associated LBP and sCD14 to cognitive function using a mediation analysis, with intracranial volume as mediator. *RIGHT*: Healthy controls showed a negative association between hippocampal volume and LBP (*b* = –0.11, *p* = .04. In the SSD patients, these associations were markedly less present (*b* = 0.005, *p* = .88). Both sCD14 and LBP were indirectly associated with lower cognitive functioning in healthy controls (LBP: *b* = –0.071, *p* = .028; sCD14: *b* = –0.213, *p* = .052), mediated by low intracranial volume (not shown in graphical abstract). These findings extend earlier studies suggesting that increased bacterial translocation may negatively affect brain volume, which indirectly impacts cognition, even in this young healthy group. If replicated, this finding stresses the importance of a healthy gut for the development and optimal functioning of the brain. Absence of these associations in the SSD group may indicate that other factors such as allostatic load, chronic medication use and interrupted educational carrier had larger impact and attenuated the relative contribution of bacterial translocation.

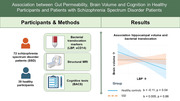

## Significant Outcomes

- We found negative associations between markers of bacterial translocation and both brain volume and cognition.
- These associations are stronger in healthy participants than in schizophrenia spectrum disorder patients.

## Limitations

- The associations we found are not as strong as we hoped, perhaps due to our young participants with relatively preserved brain volume and cognition.
- This study is observational, and cannot infer causality.

## 1 INTRODUCTION

Recent years have seen a surge of manuscripts discussing the potential influence of the gut on the brain (Cryan et al., 2019). The relation between gut and brain is multifactorial and can occur via the vagal nerve, inflammation, endocrine pathways, and more (Cryan et al., 2019). Gut permeability has often been implied as a relevant factor defining the association between gut and brain. The gut can transport compounds into the periphery through several routes, which can be divided into paracellular and transcellular pathways (Greenweld‐Van Meerveld et al., 2017; Vanuytsel et al., 2021). In a healthy gut, larger compounds such as proteins and bacteria can only move across the gut barrier through transcellular endo‐ and exocytosis (Greenweld‐Van Meerveld et al., 2017). However, factors such as chronic low‐grade inflammation, psychological stress, and diet choices may lead to disruption of the gut barrier function, increasing gut permeability (Camilleri et al., 2020; Vanuytsel et al., 2021). Consequently, gut metabolites, endotoxins like lipopolysaccharide (LPS), and even whole bacteria can leak out into the blood (i.e., bacterial translocation) (Camilleri et al., 2020; Vanuytsel et al., 2021). There, they can instigate a persistent inflammatory response, which may eventually have systemic consequences (Farre & Vicaro, 2017). Notably, increased translocation of bacteria and LPS has been associated with an array of health problems, including fatal ones such as liver cirrhosis and multi‐organ failure (Hollander & Kaunitz, 2020; Vanuytsel et al., 2021). Likewise, gut permeability has been linked to neurodegenerative and psychiatric disorders, implying that these effects may reach the brain (Maguire & Maguire, 2019).

In contrast to the plenitude of review articles on this topic, there is a paucity of data articles that actually support this hypothesized influence of the gut on the brain, and vice versa. A recent study demonstrated that gut‐microbiome features associated with decreased cognition and altered brain structure were also significantly associated with inflammatory cytokines in serum (Liang et al., 2022). Preclinical research shows that translocated bacterial peptidoglycan, derived from gut bacteria, can induce alterations in gene expression in the brain (Arentsen et al., 2017). Similarly, LPS administered peripherally can induce changes in brain cytokine and in brain neurotransmitter levels in pigs (Nordgreen et al., 2018). Research in rats has shown that LPS might cross the blood‐brain barrier through lipoprotein mediated transport (Vargas‐Caraveo et al., 2017). In mice, peripheral LPS administration is followed by microglial activation, neuroinflammation, and neurodegeneration, resulting in loss of neuronal cells in the hippocampus and cognitive impairment (Henry et al., 2010; Qin et al., 2007; Zhao et al., 2019). These microglia play an important role in brain homeostasis and plasticity (Tay et al., 2017). Aberrant microglial activation has been linked to reduced brain volume and cognitive dysfunction through neuroinflammation and increased synaptic pruning, in both different patient groups and in healthy controls (Lui et al., 2017; Nicastro et al., 2020; Opel et al., 2019). Likewise, microglial activation has been related to bacterial translocation across various neurodegenerative and psychiatric disorders (Baizabal‐Carvallo & Alonso‐Juarez, 2020; Cerovic et al., 2019; Haq et al., 2018; Yirmiya et al., 2015). As such, translocation of endotoxins and bacteria may be an important part of gut‐brain pathology, altering brain volume and function through microglial dysfunction.

How bacterial translocation occurs in a state of increased gut permeability is as of yet under debate. Increased paracellular space could potentially allow bacterial endotoxins, like LPS, to cross the gut wall (Watson et al., 2005). However, recent work in mice and rats shows that LPS mainly utilizes transcellular endocytosis, mediated via lipid rafts and cluster of differentiation 36 (CD36), and may not rely on paracellular transport (Akiba et al., 2020). Whole bacteria also likely depend on endocytosis as they are too large and too lipophilic to cross paracellularly (Farre & Vicaro, 2017; Hollander & Kaunitz, 2020). There are several biomarkers for gut permeability, assessing different pathways for crossing the gut wall (van Wijck et al., 2013; Vanuytsel et al., 2021). We will examine translocation of LPS, because of its implications in gut‐brain pathology (Nordgreen et al., 2018; Qin et al., 2007). LPS binding protein (LBP) and soluble cluster of differentiation 14 (sCD14) are used as markers of LPS in serum, and can be considered markers of endotoxemia (Stehle et al., 2012; Sun et al., 2010). Both LBP and sCD14 are pivotal for eliciting a systemic immune response in response to bacterial translocation by forming a complex with bacterial LPS (Gonzalez‐Quintela et al., 2013).

These selected markers of gut permeability also reflect a link between brain volume and bacterial translocation. LBP is associated with both decreased white matter volume and cognition in obese people (Moreno‐Navarrete et al., 2017). sCD14 is negatively related to brain volumes in people with irritable bowel syndrome (IBS) (Weaver et al., 2016), supporting the idea that increased bacterial translocation negatively affects brain volume. Similarly, recent studies in elderly found that high levels sCD14 and other inflammatory markers are correlated to low brain volume, impaired cognition and incident dementia (Fang et al., 2022; Pase et al., 2020).

The gut‐brain axis may play a role in psychiatric disorders such as schizophrenia spectrum disorders (SSD). SSD have been associated with decreased brain volume and microglial activation (Kim et al., 2017; Laskaris et al., 2016; Marques et al., 2019). Following the lead from preclinical studies, this increased microglial activation could (partly) result from increased gut permeability. Indeed, SSD patients are known to have more frequent stomach complaints, including bloating, constipation and irritable bowel syndrome (IBS) (Lee et al., 2015). Similarly, IBS patients are at higher risk for SSD and other psychiatric disorders (Lee et al., 2015). Also, SSD patients are known to have a different gut microbiome, which may lead to increased bacterial translocation (Nguyen et al., 2019; Severance et al., 2013). Some studies indeed found elevated serum markers indicative of bacterial translocation, including sCD14 and LBP, in SSD (Dzikowski et al., 2020; Safadi et al., 2021; Severance et al., 2013), although another study could not replicate this (Morch et al., 2019). Serum levels of sCD14, but not LBP, were elevated prior to schizophrenia diagnosis, which implies that it may be useful as biomarker for psychosis vulnerability (Weber et al., 2019). A study determining intestinal permeability using a mannitol absorption test, which can be viewed as a gold standard, confirmed that gut permeability was increased in schizophrenia patients (Ishida et al., 2022). Additionally, increased gut permeability was associated with decreased cognitive function, but not with positive or negative symptoms (Ishida et al., 2022). In line with this, a low‐grade inflammatory state in SSD has been associated to decreased cognitive function (Bora, 2021). This low‐grade inflammatory state could be linked to bacterial translocation. Importantly, alpha diversity (a general measure of microbiome diversity) has been associated with brain volume in SSD (Li et al., 2021).

To investigate how bacterial translocation from the gut may be related to the brain, we examined the association of LBP and sCD14 in serum with brain volumes and cognition. We expect that LBP and sCD14 will negatively correlate to brain volume. This will apply for intracranial volume (ICV), total brain volume (TBV), and hippocampal volume (HCV), which have been related to gut permeability or microglial activation (Baizabal‐Carvallo & Alonso‐Juarez, 2020; Cerovic et al., 2019; Haq et al., 2018; Laskaris et al., 2016). We expect this relation in both healthy participants, and in SSD patients. Additionally, SSD patients will have overall decreased brain volume in our selected areas (Haijma et al., 2013; Veijola et al., 2014). We expect that LBP and sCD14 levels will be increased in SSD compared to healthy participants (Dzikowski et al., 2020; Safadi et al., 2021; Severance et al., 2013) leading to decreased brain volume, but the interaction between LBP, sCD14 and brain volume will not differ per group. We expect that high LBP and sCD14 levels will be associated with low cognitive performance in both healthy controls and SSD, potentially mediated by low brain volumes.

## 2 MATERIALS AND METHODS

### 2.1 Participants

Data from 72 early‐phase SSD patients (40 psychotic disorder not otherwise specified, 25 schizophrenia, 1 schizophreniform disorder, and 6 schizoaffective disorder) and 39 healthy controls was obtained from the Simvastatin study (ClinicalTrails.gov NCT0199930) (Sommer et al., 2021; Begemann et al., 2015). Onset of psychosis in all patients was less than 3 years prior to the start of the study. Patients were recruited in both in‐ and outpatient settings in the Netherlands, between November 2013 and February 2019. All procedures followed were in accordance with the ethical standards of the Medical Research Ethics Committee Utrecht and with the Helsinki Declaration of 1964 and its later amendments. Signed informed consent was obtained from all participants included in the study.

### 2.2 Assessment of intracranial volume, brain volume, and hippocampal volume

An MRI was performed at the end of the visit, after taking a blood sample and conducting clinical rating scales (Begemann et al., 2015). MRI scans were performed using a Philips Ingenia 3.0 Tesla CX scanner with a 32‐channel SENSE head‐coil, located at the University Medical Centre Utrecht. We acquired 3D, high‐resolution T1‐weighted structural scans with a Turbo Field Echo sequence (repetition time = 10 ms, echo‐time = 4.6 ms, flip angle = 8°, reconstructed voxel size = 0.75 × 0.75 × 0.8 mm (Vanuytsel et al., 2021), field of view = 240 mm × 240 mm × 160 mm). Scan processing and assessing intracranial volumes and brain volumes was done using the Freesurfer image analysis suite, version 6.0.1 (RRID:SCR_001847) (Fischl et al., 1999; Reuter et al., 2012). Brain volumes assessed were intracranial volume (ICV), total brain volume (TBV), and hippocampal volume (HCV). TBV did not include the brain stem or the ventricles.

### 2.3 Blood sample collection and LBP/sCD14 analysis

Blood samples were obtained through venipuncture and used to quantify LBP and sCD14 levels. Both markers were measured in accordance with the protocol of the manufacturer (Human LBP DuoSet ELISA, R&D systems, Minneapolis, USA; Human sCD14 DuoSet ELISA, R&D systems, Minneapolis, USA). LBP was diluted at 1:500 and sCD14 at 1:4000. All assays were performed three times, and we used mean values of the three assessments. Variation coefficients were 4.7% for LBP and 5.1% for sCD14.

### 2.4 Cognition

Cognitive functioning was assessed using the Brief Assessment of Cognition in Schizophrenia (BACS) (Keefe et al., 2004). The BACS consists of several cognitive and motor domains (verbal memory, working memory, motor speed, verbal fluency, reasoning and problem solving, and attention and processing speed) which can be combined into a composite score. The composite score can be calculated by averaging the scores of all domains, followed by standardization. We standardized BACS scores using data gathered in a healthy population (Keefe et al., 2008). Here, a score of 0 is considered the population average.

### 2.5 Statistical analysis

Data were analyzed using Rstudio version 1.4.1106 (RRID:SCR_000432). Results are reported as average ± standard error of the mean (SEM). Group differences between SSD and healthy controls were tested using ANCOVA. Differences in brain volumes (i.e., ICV, TBV, and HCV) were corrected using age, sex, and Euler number (a measure of image quality) as covariates. Associations between brain volume with sCD14/LBP were examined using linear regression analyses and were corrected using age, sex, and BMI as covariates. In linear regression, hippocampal volume was also corrected for intracranial volume. SSD and healthy controls were analyzed together and separately, by adding an interaction term to the regression analyses. sCD14 and LBP were included in the analysis as continuous untransformed variables. We tested the mediating effect of brain volume on the relation between sCD14/LBP and cognition using a mediation analysis. Brain volumes were added as mediators in the analyses, which were performed on both groups individually. The mediation analysis determined a direct, indirect and total effect. The direct effect is the effect of sCD14/LBP on cognition. The indirect effect is the mediated effect, or the effect that sCD14/LBP have on cognition by altering brain volume. The total effect is the indirect effect and the direct effect taken together. Indirect effects are tested for significance using bootstrapping, with 1000 bootstrapped samples. Results were considered significant if *p* < .05, and were considered a trend if *p* ≤ .1. Missing data was excluded from the analysis. With our sample size of 72 SSD patients, an effect size of 0.15 would be adequately powered with β = 0.8 at α = 0.05.

## 3 RESULTS

### 3.1 Cohort characteristics

The characteristics of the selected participants are shown in Table 1. Data shown are raw data, and the statistical tests were corrected as described above. Average age in the healthy controls group was lower compared to the SSD group. As expected, ICV and TBV were significantly lower in the SSD group compared to the healthy controls. HCV did not differ significantly between the groups. BMI, LBP, and sCD14 levels did not differ between groups. LBP and sCD14 correlated to each other in the healthy controls (adjusted *R* ^2^ = 0.075, *p* = .05), but not in SSD (adjusted *R* ^2^ = 0.001, *p* = .31). We observed lower composite BACS scores in SSD (−1.34 ± 0.14) compared to healthy controls (0.1 ± 0.18) (see Table 1), indicating lower cognitive function in SSD.

**TABLE 1:** Cohort characteristics

Group	Healthy controls			SSD			Statistics
Age	24.49	±	0.79	27.74	±	0.81	*F*(1, 109) = 6.8, *p* = .01*
Number participants (% male)	39		(79%)	72		(78%)	NA
Intracranial volume (× 10^6^ mm^3^)	1.61	±	0.0289	1.53	±	0.0228	*F*(1, 109) = 4.51 *p* = .036*
Total brain volume (× 10^6^ mm^3^)	1.20	±	0.0165	1.15	±	0.0131	*F*(1, 109) = 5.66 *p* = .019*
Hippocampal volume (× 10^3^ mm^3^)	8.31	±	0.127	8.18	±	0.092	*F*(1, 108) = 0.28, *p* = .60
BMI (kg/m^2^)	23.87	±	0.72	24.23	±	0.45	*F*(1, 109) = 0.009, *p* = .42
LBP (μg/mL)	7.63	±	0.39	8.26	±	0.34	*F*(1, 109) = 1.31, *p* = .25
sCD14 (μg/mL)	2.51	±	0.16	2.51	±	0.14	*F*(1, 109) = 0.0001, *p* = .99
PANSS total score		NA		58.63	±	1.67	NA
BACS composite score	0.1	±	0.18	–1.34	±	0.14	*F*(1, 109) = 39.50, *p* = 6.93 × 10^−9^*

Abbreviations: SSD: schizophrenia spectrum disorder, LBP: lipopolysaccharide binding protein, μg: microgram, mL: milliliters, sCD14: soluble cluster of differentiation 14, mm^3^: cubic millimeter, PANSS: positive and negative symptom scale, BACS: Brief Assessment of Cognition in Schizophrenia, NA: not available.

*Indicates a significant difference between healthy controls and SSD (*p* < .05).

### 3.2 Associations of LBP and sCD14 with brain structure

**FIGURE 1:**
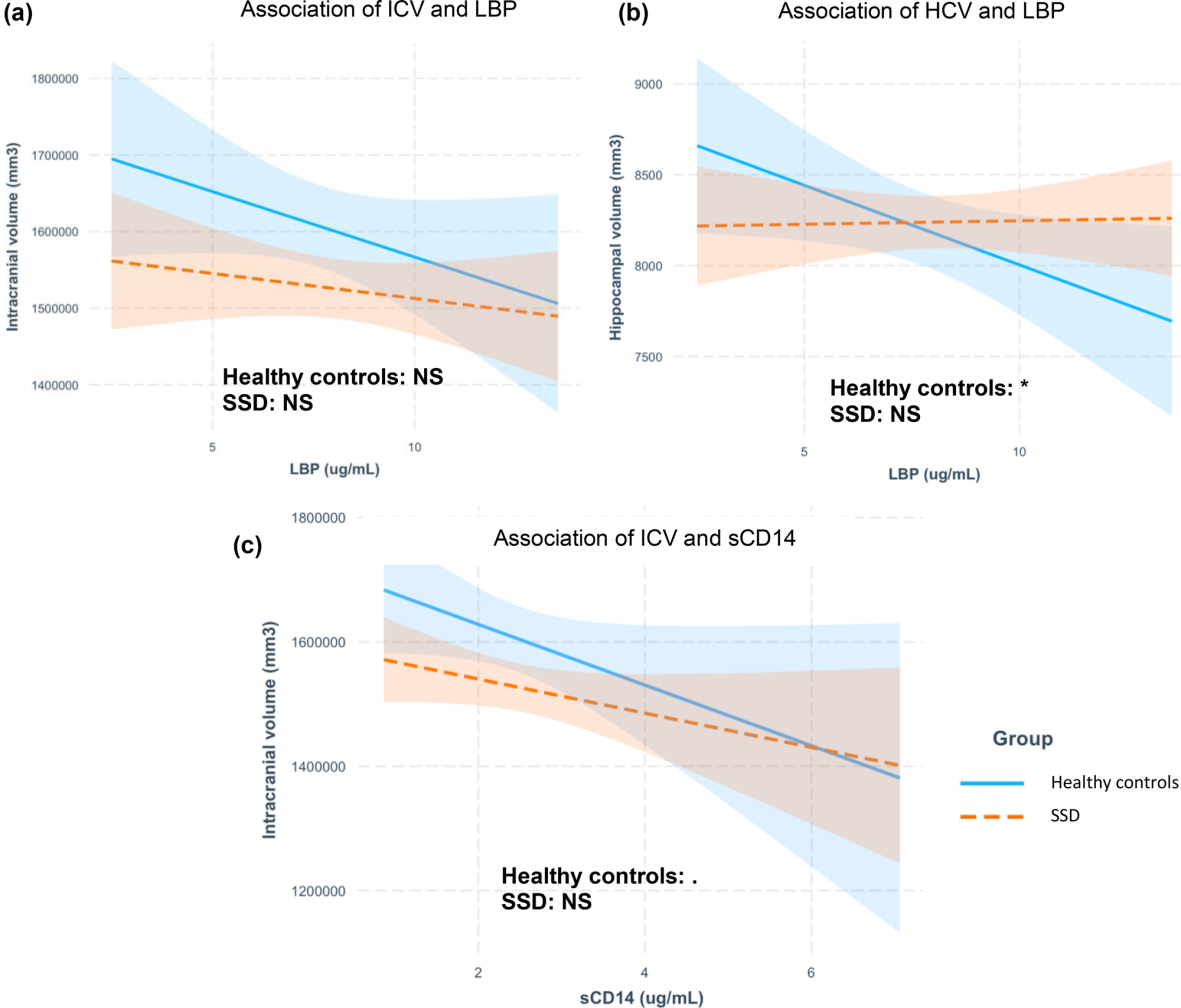
The line represents the linear model, the shade represents the standard error of the mean. In both figures, the blue line represents the healthy controls group, whereas the orange dotted line represent the SSD group. *Indicates a significant relation between ICV/HCV and LBP/sCD14 (*p* < .05). Dot indicates a trend between ICV/HCV and LBP/sCD14 (*p* ≤ .1). ICV: intracranial volume, HCV: hippocampal volume, SSD: schizophrenia spectrum disorder, LBP: lipopolysaccharide binding protein, sCD14: soluble cluster of differentiation 14, mm^3^: cubic millimeter, NS: nonsignificant.

We found nonsignificant negative associations between ICV and LBP, seen in Figure 1A (healthy controls, *b* = −0.09, *p* = .13; SSD, *b* = −0.03, *p* = .36, adjusted *R* ^2^ = 0.27). There was no correlation between TBV and LBP in either group (healthy controls, *b* = −0.06, *p* = .31; SSD, *b* = −0.04, *p* = .27, adjusted *R* ^2^ = 0.34) (data not shown). The negative correlation between HCV and LBP was significant in healthy controls, but was absent in SSD, as can be seen in Figure 1B (healthy controls, *b* = −0.11, *p* = .04; SSD, *b* = 0.005, *p* = .88, adjusted *R* ^2^ = 0.40).

There was a negative trend between sCD14 and ICV in the healthy controls, (*b* = −0.25, *p* = .07; SSD, *b* = −0.14, *p* = .11, adjusted *R* ^2^ = 0.28) (Figure 1C). There was no association between TBV and sCD14 in either group (healthy controls, *b* = −0.17, *p* = .21; SSD, *b* = −0.09, *p* = .31, adjusted *R* ^2^ = 0.39) (data not shown). Finally, HCV was not associated with sCD14 (healthy controls, *b* = −0.11, *p* = .42; SSD, *b* = −0.08, *p* = .36, adjusted *R* ^2^ = 0.39) (data not shown).

### 3.3 Association between cognition and brain structure

Total ICV positively and significantly correlated to BACS scores in the healthy controls, and ICV positively correlated on a trend level to BACS scores in the SSD group (healthy controls, *b* = 2.93 × 10^−6^, *p* = .009; SSD, *b* = 1.26 × 10^−6^, *p* = .099, adjusted *R* ^2^ = 0.29). TBV correlated significantly to BACS scores in both groups (healthy controls, *b* = 4.19 × 10^−6^, *p* = .037; SSD, *b* = 2.91 × 10^−6^, *p* = .037, adjusted *R* ^2^ = 0.32). HCV did not correlate significantly to BACS scores in either group (healthy controls, *b* = 2.82 × 10^−4^, *p* = .22; SSD, *b* = 2.85 × 10^−6^, *p* = .166, adjusted *R* ^2^ = 0.29).

### 5.4 Associations of LBP and sCD14 with cognition

BACS scores did not directly correlate with LBP, in healthy controls nor SSD (healthy controls, *b* = −0.02, *p* = .76; SSD, *b* = 0.04, *p* = .35, adjusted *R* ^2^ = 0.30). Similarly, we did not find an association between BACS and sCD14 in either group (healthy controls, *b* = −0.01, *p* = .88; SSD, *b* = 0.02, *p* = .64, adjusted *R* ^2^  = 0.27).

### 3.4 Mediation of brain structure on the relation between LBP, sCD14 with cognition

**FIGURE 2:**
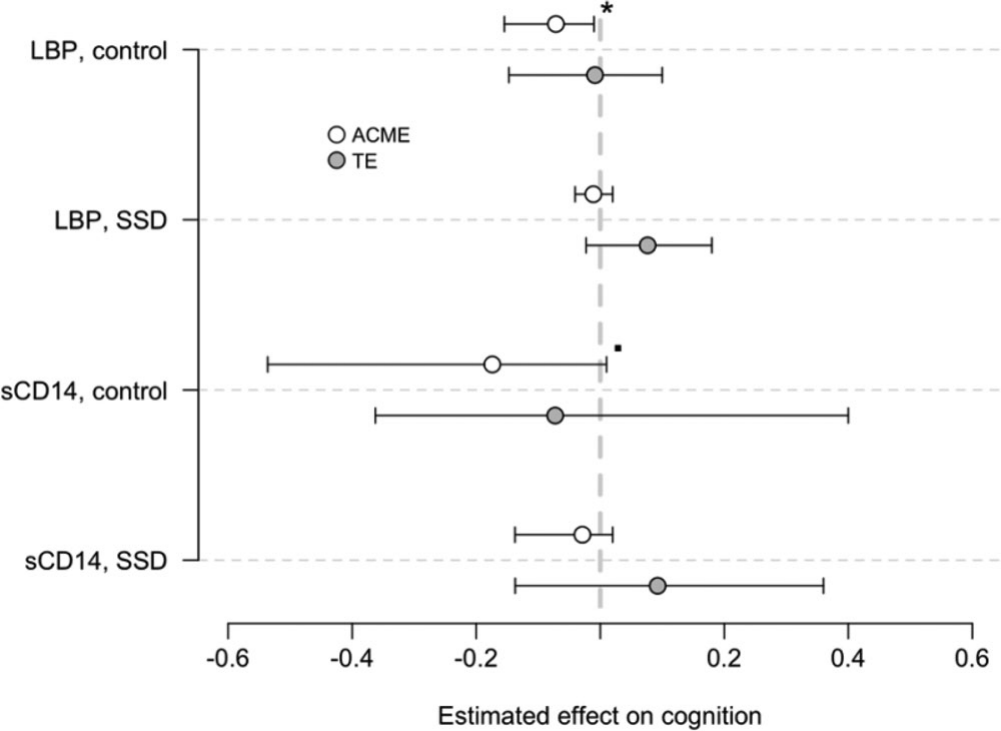
The white dots indicate the estimate ACME, the gray dots indicate the estimate TE. The error bars indicate 95% confidence intervals. The vertical, light gray, interrupted line goes through effects on cognition = 0. *Indicates a significant relation between LBP/sCD14 and cognition, mediated by ICV (*p* < .05). Dot indicates a trend between LBP/sCD14 and cognition, mediated by ICV (*p* < .10). ICV: intracranial volume, SSD: schizophrenia spectrum disorder, LBP: lipopolysaccharide binding protein, sCD14: soluble cluster of differentiation 14, ACME: average causal mediated effects, TE: total effects.

The mediation analysis (see methods section 2.4 for details) showed a negative effect of LBP on BACS mediated by decreased ICV in the healthy controls, but not in SSD (healthy controls, *b* = −0.071, *p* = .028; SSD, *b* = −0.011, *p* = .37), and is seen in Figure 2. The total effect was not significant in either group (healthy controls, *b* = −0.008, *p* = .74; SSD, *b* = 0.076, *p* = .132). Similarly, we found a negative trend of sCD14 on BACS scores mediated by decreased ICV in healthy controls, but not in SSD (healthy controls, *b* = −0.174, *p* = .068; SSD, *b* = −0.029, *p* = .33). Total effects of sCD14 on BACS scores mediated by ICV was not significant in either group (healthy controls, *b* = 0.073, *p* = .77; SSD, *b* = 0.093, *p* = .42). We found no effects of LBP or sCD14 on cognition when mediated by other measures of brain volume (see Appendix 1).

## 4 DISCUSSION

We examined associations between LBP and sCD14, brain volumes and cognition to determine if bacterial translocation markers may affect structure and function of brain volumes in a young healthy cohort (*n* = 39) and early‐phase SSD patients (*n* = 72). As expected, the SSD group had lower intracranial volume (ICV) and total brain volume (TBV), but hippocampal volume (HCV) was similar. In extension, cognition was lower in the SSD group. We found that, in healthy controls, sCD14 negatively correlated to ICV, and that LBP negatively correlated to HCV. Despite not finding a direct effect of bacterial translocation markers on cognitive function, LBP and sCD14 did have an indirect effect on cognitive functioning in healthy controls through decreasing ICV. Unexpectedly, there were no differences in LBP or sCD14 between groups. Additionally, we did not observe any associations between bacterial translocation markers and brain volume or cognition in the SSD group.

We hypothesized that bacterial translocation would relate to decreased intracranial volume, total brain volume and hippocampal volume. Indeed, LBP correlated significantly with HCV in healthy controls, and sCD14 correlated on a trend level with ICV in healthy controls. Similar to our results, reduced TBV and HCV was found in healthy older subjects with increased sCD14 (Pase et al., 2020). Obese people have been shown to have impaired cognition and low brain volume, especially in the hippocampus, which has also been linked to increased gut permeability and neuroinflammation (Olsthoorn et al., 2021). The link between sCD14 with ICV in our study suggests that the relation between brain and bacterial translocation originates at least partly during development, as ICV is already determined at a young age (Haijma et al., 2013; Pfefferbaum et al., 1994). Importantly, intestinal permeability may be mostly stable throughout life, although studies in children are limited (Wilms et al., 2020). Microglia have been shown to be sensitive to activation during adolescence, and disturbances during this period can lead to alterations in synaptic density and cognitive function in later life (Schalbetter et al., 2022). As such, our findings may reflect the negative impact of intestinal permeability on the developing brain.

The impact of the bacterial translocation on cognition seems to be relatively low in this cohort. Neither LBP nor sCD14 had a direct correlation with BACS scores in either group. However, they were indirectly negatively correlated to BACS scores in the healthy controls when mediated by decreased ICV. That this observed effect was specifically indirect, but not direct, is also consistent with a developmental origin for the relation between brain and bacterial translocation. Our findings are congruent with findings on sCD14 in an older cohort (Pase et al., 2020). The significance level of sCD14 was modest, which may be a consequence of the relatively young age and preserved cognition of our participants. In our cohort, low brain volume is associated with low cognition. This correlation between brain volume in these regions and cognition is well established (Van Loenhoud et al., 2018; Vibha et al., 2018).

Unexpectedly, there were no significant differences in LBP or sCD14 levels between healthy controls and SSD patients, thus refuting the hypothesis that there is more bacterial translocation in this patient group. Our study is in line with some previous findings of LBP levels in SSD that did not find an increase in comparison to controls (Severance et al., 2013), while Morch et al. (2019) even found decreased levels of LBP in SSD patients. Findings on sCD14 in SSD have been mixed, with some studies finding increased levels of sCD14 (Severance et al., 2013; Safadi et al., 2021; Weber et al., 2019), whereas others did not (Morch et al., 2019). sCD14 is not only a marker for bacterial translocation, but also for monocyte activation (Shive et al., 2016). Some studies report monocyte activation in SSD, although this is not a consistent finding (Moody & Miller, 2018; Garcia‐Rizo et al., 2019). Therefore, increased sCD14 levels in SSD found by other studies might have reflected monocyte activation unrelated to increased bacterial translocation. In support of this argument, LBP and sCD14 were not correlated in SSD patients in our sample, but were correlated in the healthy controls. This inconsistency in literature, together with negative findings from the present study, does not support a strong role for increased bacterial translocation in SSD.

We could not find associations between brain volumes and LBP or sCD14 in SSD patients, nor mediating effects on cognition. In our sample, SSD patients had decreased ICV and TBV compared to controls, replicating previous findings (Kim et al., 2017; Haijma et al., 2013; Veijola et al., 2014). This suggests that SSD‐specific pathophysiological processes may lead to an extra decrease in brain volumes, potentially reducing the relative effects of bacterial translocation on the brain. For example, chronic use of antipsychotics is associated with a decrease in brain volume (Haijma et al., 2013). Additionally, increased allostatic load, a result of severe and chronic stressors, is also known to affect brain volumes in both SSD and healthy controls (Chiappelli et al., 2017). As SSD patients are known to have significantly more allostatic load than healthy controls (Chiappelli et al., 2017), these factors may be more pronounced than gut‐brain interactions in the patient group. Similarly, the correlation between markers of bacterial translocation and cognitive scores was absent in the SSD group. Other studies have found similar results, suggesting that serum markers of bacterial translocation are not related to cognition in SSD (Morch et al., 2019; Tanaka et al., 2017).

This study is subject to several limitations. First, our SSD group is young, with relatively preserved cognition. Second, we examine bacterial translocation, which is only one part of gut permeability. Whether bacteria and their endotoxins cross the gut transcellularly and/or paracellularly remains unclear (Hollander & Kaunitz, 2020). Use of other markers may help differentiate between pathways of crossing the gut, although current in vivo methods are not able to fully distinguish between pathways (Schoultz & Keita, 2020). The tight junction protein zonulin, a serum marker of gut permeability, may be useful, although commercially available assays may not measure zonulin properly (Schoultz & Keita, 2020). Alternatively, measuring the concentration of orally ingested probes in urine is a demanding, but more direct and probably more accurate method of assessing intestinal permeability (van Wijck et al., 2013). Inclusion of other markers concerning inflammatory pathways implicated in SSD pathology may help to contextualize the role of bacterial translocation in SSD. A potential candidate would be the toll‐like receptor 4 pathway, which includes sCD14 and LBP, and is implicated in SSD (Gonzalez‐Quintela et al., 2013; García‐Bueno et al., 2016). Third, this study had a power of 0.8 to detect effect sizes of 0.15. The detected effect sizes were sometimes smaller than 0.15, for which are study was underpowered. Finally, because this study is observational, we are unable to infer any causality.

## 5 CONCLUSION

In conclusion, we found several associations between serum markers of bacterial translocation, LBP and sCD14, and the decrease in intracranial volume and hippocampal volume in healthy young participants. This supports the hypothesis that increased gut permeability negatively impacts brain development. In our healthy group, LBP and sCD14 were indirectly associated with decreased cognition, with intracranial volume as a mediator. These correlations were not observed in SSD patients, perhaps because other factors that were absent in controls, such as disease effects, medication effects, high stress and trauma levels, had stronger influences on brain volumes in this group. Future research is needed to replicate and extend these findings in larger groups.

## FUNDING

This work was supported by the Stanley Medical Research Institute (grant number 18T‐004) and ZonMW (Netherlands Organisation for Health Research and Development; grant number 636320010). These institutions had no further involvement in this study.

## CONFLICT OF INTEREST STATEMENT

All authors report no biomedical financial interests or potential conflicts of interest.

### PEER REVIEW

The peer review history for this article is available at https://publons.com/publon/10.1002/brb3.3011.

## ETHICS STATEMENT

All procedures followed were in accordance with the ethical standards of the Medical Research Ethics Committee Utrecht and with the Helsinki Declaration of 1964 and its later amendments. Signed informed consent was obtained from all participants included in the study.

## Supporting information

Appendix 1: Mediation analysis

## Data Availability

The data that support the findings of this study are available from the corresponding author upon reasonable request.
