# Supplementary material for: Association between gut permeability, brain volume, and cognition in healthy participants and patients with schizophrenia spectrum disorder
Source: Brain Behav. 2023 Apr 24;13(6):e3011. doi: 10.1002/brb3.3011 (PMC10275537; doi:10.1002/brb3.3011)
Supplement: Supplementary file 1 — Appendix 1: Mediation analysis [file BRB3-13-e3011-s001.docx]

# **Appendix 1: Mediation analysis**

*4 tables showing the effect of LBP and sCD14 on BACS scores in both HEALTHY CONTROLS and SSD, mediated via brain volume. Each table represents one brain region. * indicates p<0.05 and . indicates p =/< 0.1.*

*Abbreviations: ICV: intracranial volume, TBV: Total brain volume, HCV: Hippocampal volume, CI LL: Confidence interval lower level, CC UL: Confidence interval upper level, LBP: Lipopolysaccharide binding protein, sCD14: soluble cluster of differentiation 14, SSD: Schizophrenia spectrum disorders, BACS: brief assessment of cognition in schizophrenia, ACME: Average causal mediated effects, TE: Total effects.*

**Table A1 ICV mediation**

|  |  |  |  | Estimate | 95% CI LL | 95% CI UL | p-value |
| --- | --- | --- | --- | --- | --- | --- | --- |
| ICV | **SSD** | Healthy controls | ACME | -0.071 | -0.151 | -0.01 | 0.028 * |
|  |  |  | TE | -0.008 | -0.146 | 0.09 | 0.736 |
|  |  | SSD | ACME | -0.011 | -0.042 | 0.02 | 0.366 |
|  |  |  | TE | 0.076 | -0.023 | 0.18 | 0.132 |
|  | **sCD14** | Healthy controls | ACME | -0.174 | -0.524 | 0.01 | 0.068 . |
|  |  |  | TE | 0.073 | -0.316 | 0.42 | 0.766 |
|  |  | SSD | ACME | -0.029 | -0.129 | 0.02 | 0.33 |
|  |  |  | TE | 0.093 | -0.124 | 0.35 | 0.42 |

**Table A2 TBV mediation**

|  |  |  |  | Estimate | 95% CI LL | 95% CI UL | p-value |
| --- | --- | --- | --- | --- | --- | --- | --- |
| TBV | **SSD** | Healthy controls | ACME | -0.032 | -0.112 | 0.01 | 0.18 |
|  |  |  | TE | -0.008 | -0.148 | 0.09 | 0.76 |
|  |  | SSD | ACME | -0.014 | -0.049 | 0.02 | 0.39 |
|  |  |  | TE | 0.076 | -0.018 | 0.18 | 0.15 |
|  | **sCD14** | Healthy controls | ACME | -0.084 | -0.369 | 0.03 | 0.22 |
|  |  |  | TE | 0.073 | -0.372 | 0.41 | 0.84 |
|  |  | SSD | ACME | -0.022 | -0.142 | 0.02 | 0.38 |
|  |  |  | TE | 0.093 | -0.131 | 0.35 | 0.40 |

**Table A3 HPV mediation**

|  |  |  |  | Estimate | 95% CI LL | 95% CI UL | p-value |
| --- | --- | --- | --- | --- | --- | --- | --- |
| HPV | **SSD** | Healthy controls | ACME | -0.047 | -0.144 | 0.06 | 0.35 |
|  |  |  | TE | -0.008 | -0.158 | 0.09 | 0.71 |
|  |  | SSD | ACME | -0.004 | -0.038 | 0.03 | 0.81 |
|  |  |  | TE | 0.076 | -0.022 | 0.17 | 0.13 |
|  | **sCD14** | Healthy controls | ACME | -0.033 | -0.214 | 0.07 | 0.54 |
|  |  |  | TE | 0.073 | -0.348 | 0.40 | 0.76 |
|  |  | SSD | ACME | -0.047 | -0.160 | 0.01 | 0.16 |
|  |  |  | TE | 0.093 | -0.133 | 0.36 | 0.40 |

|  |  |  |
| --- | --- | --- |
